# *Arabidopsis* Bax Inhibitor-1 inhibits cell death induced by pokeweed antiviral protein in *Saccharomyces cerevisiae*

**DOI:** 10.15698/mic2015.02.190

**Published:** 2015-02-02

**Authors:** Birsen Çakır, Nilgun E. Tumer

**Affiliations:** 1 Biotechnology Center for Agriculture and the Environment and the Department of Plant Biology and Pathology, Rutgers University, New Brunswick, NJ 08901-8520, USA.; 2 Department of Horticulture, Faculty of Agriculture, Ege University, Izmir, Turkey.

**Keywords:** ribosome inactivating proteins (RIPs), pokeweed antiviral protein (PAP), Arabidopsis thaliana Bax Inhibitor-1, apoptotic-like cell death

## Abstract

Apoptosis is an active form of programmed cell death (PCD) that plays critical roles in the development, differentiation and resistance to pathogens in multicellular organisms. Ribosome inactivating proteins (RIPs) are able to induce apoptotic cell death in mammalian cells. In this study, using yeast as a model system, we showed that yeast cells expressing pokeweed antiviral protein (PAP), a single-chain ribosome-inactivating protein, exhibit apoptotic-like features, such as nuclear fragmentation and ROS production. We studied the interaction between PAP and AtBI-1 (*Arabidopsis thaliana* Bax Inhibitor-1), a plant anti-apoptotic protein, which inhibits Bax induced cell death. Cells expressing PAP and AtBI-1 were able to survive on galactose media compared to PAP alone, indicating a reduction in the cytotoxicity of PAP in yeast. However, PAP was able to depurinate the ribosomes and to inhibit total translation in the presence of AtBI-1. A C-terminally deleted AtBI-1 was able to reduce the cytotoxicity of PAP. Since anti-apoptotic proteins form heterodimers to inhibit the biological activity of their partners, we used a co-immunoprecipitation assay to examine the binding of AtBI-1 to PAP. Both full length and C-terminal deleted AtBI-1 were capable of binding to PAP. These findings indicate that PAP induces cell death in yeast and AtBI-1 inhibits cell death induced by PAP without affecting ribosome depurination and translation inhibition.

## INTRODUCTION

Ribosome inactivating proteins (RIPs) that are toxins isolated from plants, fungus, or bacteria catalytically inactivate eukaryotic as well as prokaryotic ribosomes by removing single adenine residues from the universally conserved sarcin/ricin loop (SRL) of the large rRNA 1,2,3,4. In addition to rRNA *N*-glycosidase activity, RIPs have broad spectrum antiviral activity against RNA and DNA from plant and animal viruses 5,4.

Pokeweed antiviral protein (PAP), a single chain type I RIP, isolated from leaves of pokeweed plants (*Phytolacca americana*), removes specific adenine and guanine residues from the SRL 1,6,7. This enzymatic activity interferes with the binding of eEF-2 (elongation factor 2) thereby inhibiting protein synthesis at the translocation step 8,9. PAP has antiviral activity against animal and plant viral pathogens including HIV, poliovirus, herpes simplex virus, influenza, potato virus X, and BMV 10,11,12,13,14. Hudak *et al.* 7,15 demonstrated that PAP could also inhibit translation of mRNAs and viral RNAs that are capped by binding to the cap structure and were depurinating the RNAs downstream of the cap. It has been reported that the antiviral activity of PAP can be separated from rRNA depurination 16. These results suggested that PAP might interfere with virus replication by a mechanism other than host ribosome inactivation. One possible mechanism is that RIPs might target not only the SRL but also the nucleic acids of invading pathogens. Wang and Tumer 17 showed that PAP cleaved double stranded supercoiled DNA using the same active site required for ribosome depurination. Similar activity of other RIPs on supercoiled double-stranded DNA templates was observed with dianthin, gelonin, cinnamomin and saporin 18,19,20.

Besides inhibition of protein synthesis, RIPs are able to induce apoptosis in different cells 21,22,23,24,25. Griffiths *et al.* 26 demonstrated that ricin and abrin induced apoptosis in mammalian cells. Many other bacterial as well as plant toxins were also found to induce apoptosis in mammalian cells 21,22,23,25,27,28,29. Work from our laboratory has shown that the precursor form of the A chain of ricin (pre-RTA) in yeast cells induced the onset of apoptotic markers such as nuclear fragmentation, chromatin condensation, and accumulation of reactive oxygen species 30. The ability to depurinate ribosomes and inhibit translation does not always correlate with ricin-mediated cell death 30. The cell death induced by the RIPs, such as ricin, modeccin, diphtheria toxin and pseudomonas toxin involves caspases 31,32. In addition, trichosanthin, a type I RIP, has been shown to induce apoptosis by high levels of ROS production in human choriocarcinoma cells 33.

Apoptosis is co-regulated by the conserved family of Bcl-2 related proteins, which includes both antiapoptotic (e.g., Bcl-2 and Bcl-XL) and proapoptotic (e.g., bax and bak) members. A human Bax inhibitor-1 (BI-1) gene was isolated as a suppressor of bax induced cell death in yeast. BI-1 is evolutionary conserved ER protein that suppresses cell death in plants, yeast and animal cells 34. Recently, a yeast BH3 domain containing protein (Ybh3p) was identified and regulates the mitochondrial pathway of apoptosis 33. Interestingly, overexpression of Ybh3p sensitizes yeast cells to apoptotic stimuli, while its knockout reduces cell death 33. Although no homologs of Bcl-2 family proteins have been identified in plants, BI-1 is widely conserved in organisms, including *Caenorhabditis elegans* and *Xenopus leavis* 34. Subsequently, BI-1 homologs from plants have been isolated 34,35,36,37,38,39. AtBI-1, a plant homologue of BI-1 from *Arabidopsis thaliana *suppresses Bax and H_2_O_2_ mediated cell death in yeast, animal, and plant cells 40,41,42. AtBI-1 is mainly localized in the ER, contains 6 or 7 transmembrane domains with highly conserved C-terminal region that is required for the suppression of cell death 41. Overexpression of AtBI-1 was shown to suppress cell death induced by biotic and abiotic stresses 36,41,42. Overexpression of Bax in plant cells causes ROS generation, organelle disruption, and ion leakage from cells 44,45 and AtBI-1 prevents ion leakage, but not ROS generation, when overexpressed together with Bax in *Arabidopsis* 43. Recently, Watanabe and Lam 46 demonstrated that AtBI-1 played an important role in attenuation of ER stress-induced cell death 46,47. Another plant BI-1 homologue in*Capsicum annuum* has been shown to be induced under various abiotic stresses including high salinity, heavy metal stresses and ABA 48. Moreover, it was demonstrated that AtBI-1 interacts with calmodulin (CAM) and the cell death suppression activities of AtBI-1 in plant cells are mediated by modulation of ion homeostasis. In addition, Oshimo *et al.* 49 reported that BI-1 requires a functional electron transport chain for cell death suppression in yeast 49.

Although these reports indicate that BI-1 regulates cell death mechanism in animals, yeast, plants, the molecular mechanism by which AtBI-1 inhibits cell death is still unclear. The cell-death induced by plant toxins that inhibit protein synthesis is also not well understood. Their mode of action on cell death needs to be studied further.

Here, we examined the ability of PAP to induce cell death in yeast cells. Yeast expressing PAP displayed apoptosis-like features such as nuclear fragmentation and ROS production. We then studied the interaction between PAP and AtBI-1 for a possible effect of AtBI-1 on PAP induced cell death in yeast. Our results showed that AtBI-1 inhibited cell death induced by PAP in yeast. PAP was able to depurinate the ribosomes and inhibit translation in the presence of AtBI-1. To our knowledge, this is the first report demonstrating that PAP induces cell death in yeast and AtBI-1 inhibits PAP-mediated cell death independent of ribosome depurination and translation inhibition.

## RESULTS

### PAP expression in yeast causes cell death and AtBI-1 expression attenuates PAP induced cell death

**Figure 1:**
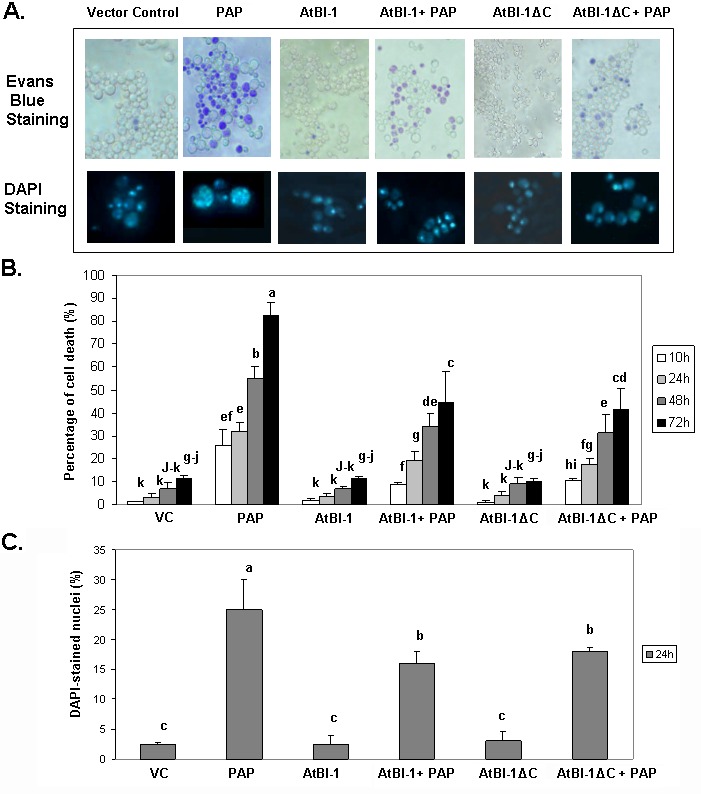
FIGURE 1: Analysis of cell death and nuclear fragmentation in yeast cells expressing PAP, AtBI-1 and AtBI-1∆C. **(A)** Cells were stained with Evans Blue or DAPI at 24 h after induction and visualised using Zeiss Axiovert 200 inverted microscope (magnification, X 40) nuclei are shown enlarged 40 times relative to the yeast cells. **(B)** The percentage of the cell death at different hours after induction were quantified and are represented as the means ± standard deviation (n=3). **(C)** DAPI stained nuclei at 24 h post-induction were quantified and are represented as the means ± standard deviation (n=3). At least 100 cells were counted per experiment. The results represent three independent experiments. VC - vector control. Columns are statistically different according to ANOVA (P < 0.001) followed by a post-hoc Fisher's Least Significant Difference (LSD) test.

RIPs such as ricin and abrin are able to induce apoptosis in a wide variety of cells and cell lines 21. Though many studies have been reported on toxin-induced apoptosis, we have very little knowledge of the mechanism of RIP-induced apoptosis. Because of the extreme toxicity of PAP, a type I RIP, in plant cells, the yeast, *S. cerevisiae*, has been used and demonstrated to be a powerful tool for genetic and biochemical characterization of PAP 50. When PAP cDNA was expressed in yeast under the control of*GAL1* promoter, cell growth was inhibited 50. Previous results indicated that ribosome depurination activity of PAP does not always correlate with its translation inhibition activity and is not sufficient for cytotoxicity 51. In this study, we investigated the ability of PAP to induce cell death in yeast. PAP cDNA was transformed into yeast. Cells were grown in glucose containing medium, then switched to fresh medium containing galactose to induce expression. At different times after induction, cells were recovered from liquid medium by centrifugation and cell viability was determined on the basis of the ability to take up Evans blue dye. Fig. 1A presents results from a representative experiment, showing an increase in the number of cells taking up Evans blue dye in cultures of PAP transformants in galactose containing medium in a time dependent manner. By 24 h post-induction, very few cells survive. These results were confirmed using control cells harboring an empty plasmid which remained mostly dye negative indicating more viable cells (Fig. 1A).

*Arabidopsis *Bax Inhibitor-1 (AtBI-1), a plant antiapoptotic protein is able to suppress Bax mediated cell death in plants, as well as in yeast cells 52,41. To determine if AtBI-1 affects PAP-induced cell death, we cloned full length AtBI-1 cDNA upstream of the V5 tag in pYES 2.1 (Topo Cloning Kit, Invitrogen) vector, in which the expression is under the control of yeast *GAL1* promoter. W303 yeast strain has been co-transformed with shuttle vectors harboring PAP and AtBI-1 cDNAs, grown in glucose containing medium, switched to galactose containing medium for induction before staining with Evans blue. As shown in Fig. 1A, yeast cells expressing PAP were stained with Evans blue dye, in contrast to cells expressing AtBI-1, which remained mostly dye negative. Yeast co-expressing AtBI-1 and PAP showed more Evans blue dye excluding cells, indicating an increase in cell viability (Fig. 1A).

Previous studies demonstrated that the C-terminal region of AtBI-1 is necessary for the inhibition of Bax induced cell death in yeast 43,42. The deletion of the last 14 amino acids completely abolished cell death suppression ability of AtBI-1 43. To determine the functional domain of AtBI-1 responsible for reduced cytotoxicity of PAP, we produced AtBI-1 C-terminal truncation mutant called AtBI-1∆C (last 23 aa - 224 to 247 - were deleted) and subcloned it into pYES 2.1 vector upstream of V5 epitope. We next co-transformed W303 yeast strain with AtBI-1∆C and PAP containing plasmids, grew in glucose containing medium then switched to galactose medium for induction. Cells were stained with Evans blue to test the possible effect of C-terminal deletion of AtBI-1 on cell viability in the presence of PAP. As shown in Fig. 1A, viability of cells expressing PAP and AtBI-1 was similar to cells expressing PAP and AtBI-1∆C, suggesting that the deletion of C-terminal region did not affect the ability of AtBI-1 to suppress the cytotoxicity of PAP.

Apoptotic cell death is characterized by chromatin condensation, nuclear fragmentation and DNA fragmentation in mammalian and yeast cells 53,54,55. We examined nuclear fragmentation in those cells to further characterize cell death process induced by PAP. Staining PAP expressing yeast cells with DAPI revealed nuclear fragmentation 24 h after induction, whereas PAP and AtBI-1 co-transformed yeast cells showed a significant decrease in the number of cells with nuclear fragmentation (Fig. 1A and 1C). Chromatin condensation and nuclear fragmentation had been already observed in yeast 56,57. After overexpression of PAP, cells showed accumulation of DAPI staining within the area of the nucleus to the appearance of multiple stained regions within a single cell. No nuclear fragmentation was observed in cells expressing AtBI-1 or vector control. These data further confirmed that PAP induces cell death in yeast and AtBI-1 expression attenuates PAP induced cell death. In addition the mutation at C-terminal domain of AtBI-1 does not affect the death suppression ability of AtBI-1 when expressed with PAP.

### PAP induces ROS production in yeast

**Figure 2:**
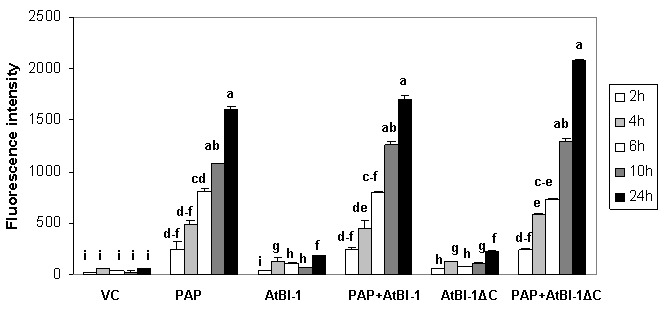
FIGURE 2: ROS generation in cells expressing PAP and AtBI-1. The amount of H_2_O_2_ production was quantified using DCDHF-DA. The results are represented as the means ± standard deviation (n=3). VC - vector control. The results represent three independent experiments. Columns are statistically different according to ANOVA (P < 0.001) followed by a post-hoc Fisher's Least Significant Difference (LSD) test.

The accumulation of ROS is one of apoptotic cell death features involved in many forms of cell death 58in animals, yeast and plants 55,59,60. To determine whether ROS generation was involved in PAP induced cell death, we quantified intracellular ROS production by using DCDHF-DA oxidation as a marker to measure intracellular levels of H_2_O_2_. As shown in Fig. 2, H_2_O_2_ level was increased in cells expressing PAP up to 24 h post-induction, which correlated well with cell death. In contrast, we did not observe any increase in the level of H_2_O_2_ up to 24 h post-induction in cells expressing vector control. To determine whether cell death inhibitory activity of AtBI-1 was accompanied by the inhibition of ROS generation, cells expressing PAP and AtBI-1 were also examined. Measurement of ROS generation did not reveal any decrease in H_2_O_2_ level in cells expressing PAP and AtBI-1 or AtBI-1∆C, indicating AtBI-1 function as a negative regulator of cell death independent of ROS accumulation, downstream of ROS or both. In addition, the C-terminal deletion of AtBI-1 did not affect H_2_O_2_ production in cells expressing PAP.

### AtBI-1 reduces PAP toxicity in yeast

**Figure 3:**
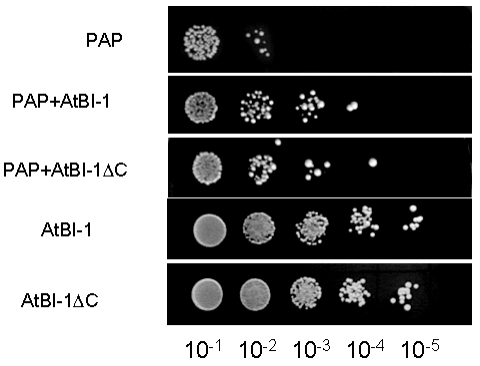
FIGURE 3: Expression and functional characterization of AtBI-1. Yeast cells containing AtBI-1 or PAP were grown in SD-U-L/raf overnight then diluted into SD-U-L/galactose for induction at 4 h for PAP and AtBI-1, then serial dilution were spotted on glucose containing plates and incubated for 48 h at 30°C.

PAP expression is toxic to yeast cells. To investigate whether AtBI-1 overexpression inhibits PAP toxicity in yeast cells, we tested its ability to rescue against PAP toxicity by examining cell viability. We transformed yeast cells with AtBI-1 and PAP, then plated onto galactose selective media to induce PAP and AtBI-1 expression. The empty plasmid has been used as a negative control. We then investigated irreversible growth inhibition by carrying out cell viability assay. Yeast cells transformed with PAP and AtBI-1 have been induced in liquid selective medium containing galactose (different induction times were tried out), then they were plated on medium containing glucose. As shown in Fig. 3, PAP expression reduced cell viability at 4 h post-induction, whereas yeast cells expressing PAP in the presence of AtBI-1 slightly restored growth of colonies, indicating reduction of PAP toxicity. We conclude that AtBI-1 is capable of rescuing yeast cells from PAP toxicity.

Cells expressing PAP and AtBI-1∆C restored growth of colonies at 4 h as compared to cells expressing only PAP. Interestingly, AtBI-1∆C could protect yeast cells from cytotoxicity of PAP as well as the full length AtBI-1. These results indicate that C-terminus of AtBI-1 is not critical for reducing the cytotoxicity of PAP.

### PAP depurinates ribosomes in the presence of AtBI-1

**Figure 4:**
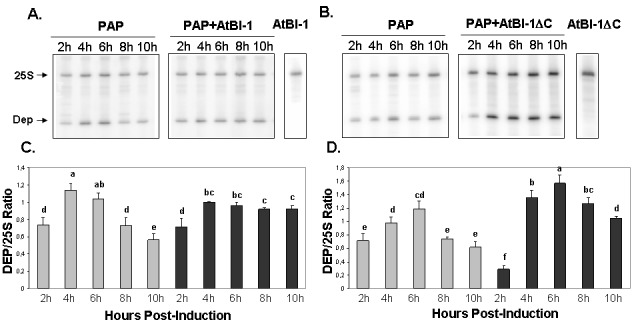
FIGURE 4: Ribosome depurination in yeast cells. Primer extension analysis in yeast cells expressing PAP and AtBI-1 **(A)** or PAP and AtBI-1∆C **(B)** using two different end labeled primers, the depurination primer (Dep) was used to measure the extend of depurination and the 25S rRNA primer (25S) was used to measure the amount of 25S rRNA **(C)** and ratio of Dep/25S **(D)**. The results represent three independent experiments. Columns are statistically different according to ANOVA (P < 0.001) followed by a post-hoc Fisher's Least Significant Difference (LSD) test.

To determine if the reduction in PAP toxicity in the presence of AtBI-1 is due to reduced depurination of ribosomes, we examined ribosome depurination using a primer extension assay. After inducing PAP and AtBI-1 expression in galactose containing media in yeast, we isolated total RNA and examined depurination by using a previously described dual primer extension assay 61. As shown in Fig. 4, ribosomes were depurinated in yeast cells expressing PAP and AtBI-1 at similar levels as in cells expressing PAP alone. Depurination in cells expressing PAP peaks by 4 h and decreases gradually up to 10 h post-induction. Similarly, PAP depurinated ribosomes when it was co-expressed with AtBI-1 (Fig. 4A).

As shown in Fig. 4A and C, depurination decreased in cells expressing PAP by 10 h post-induction. Ribosomes were depurinated in cells co-expressing PAP and AtBI-1. However, depurination did not decrease in these cells, possibly because these cells did not die unlike cells expressing PAP alone (Fig. 4C and D). The deletion of the C-terminal domain of AtBI-1 did not reduce the ribosome depurination activity of PAP as compared to that of the full length AtBI-1 in the presence of PAP. Moreover, cells expressing PAP and AtBI-1 survive better, even though their ribosomes are depurinated.

### AtBI-1 does not affect translation inhibition by PAP

**Figure 5:**
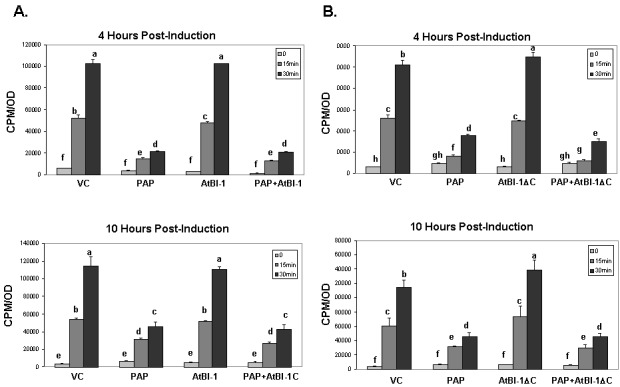
FIGURE 5: Analysis of total translation in yeast cells. **(A)** Total translation in yeast cells expressing PAP and AtBI-1. **(B)** Total translation in yeast cells expressing PAP and AtBI-1∆C. Yeast cells were grown in SD-L-U-Met and 2% glucose overnight then switched to 2% galactose containing media to induce the expression. At time 0, 35 methionine was added to cells growing on galactose which expressed PAP or AtBI-1 and incorporation of 35 methionine was determined at the indicated times. Each point was repeated in duplicate. The results represent three independent experiments. VC - vector control. Columns are statistically different according to ANOVA (P < 0.001) followed by a post-hoc Fisher's Least Significant Difference (LSD) test.

To determine whether reduction of PAP toxicity is related to reduction in translation inhibition activity of PAP, we examined total translation in yeast cells expressing PAP and AtBI-1 compared with cells expressing PAP alone and vector control. Total translation was examined by 35 methionine incorporation at 0, 4, 6, 10 h post-induction. As shown in Fig. 5A, in yeast cells expressing PAP and AtBI-1 at 4 h post-induction, translation was inhibited at the same level as with PAP alone, whereas in cells expressing AtBI-1, translation increased gradually over time. These results indicate that total translation is inhibited in cells co-expressing PAP and AtBI-1 at a similar level as in cells expressing PAP alone, indicating that AtBI-1 expression does not have any effect on the translation inhibition activity of PAP.

As shown in Fig. 5B, total translation was significantly inhibited in cells expressing PAP and AtBI-1∆C at a similar level as PAP alone, whereas total translation was not inhibited in cells expressing AtBI-1 mutant or vector control. These results indicate that the reduction in the cytotoxicity of PAP in the presence of full length or C-terminally deleted AtBI-1 is not due to a decrease in the translation inhibitory activity of PAP.

### AtBI-1 and PAP mRNAs are upregulated in yeast co-expressing AtBI-1 and PAP 

**Figure 6:**
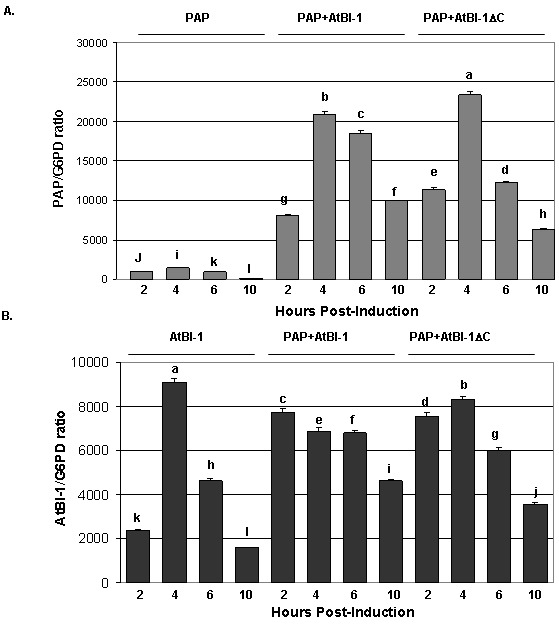
FIGURE 6: Real-time PCR analysis of mRNA levels in yeast cells. **(A) **Analysis of *PAP* mRNA in yeast cells expressing AtBI-1. **(B)** Analysis of *AtBI-1* mRNA in yeast cells expressing PAP. Cells were grown on galactose for the hours indicated. The mRNA levels for the genes were normalized to *G6PD* mRNA using the ∆∆CT method from Applied Biosystems. The results represent three independent experiments. Columns are statistically different according to ANOVA (P < 0.001) followed by a post-hoc Fisher's Least Significant Difference (LSD) test.

To determine if the reduction in the cytotoxicity of PAP is due to a decrease in PAP expression we isolated total RNA and examined PAP mRNA expression pattern in yeast cells co-expressing PAP and AtBI-1 at various times post-induction (Fig. 6). PAP mRNA level in yeast expressing AtBI-1 was upregulated compared to yeast expressing PAP alone (Fig. 6A). PAP transcript level increased by 11-fold compared to that of PAP alone by 6 h post-induction, and then decreased up to 10 h post-induction. AtBI-1 mRNA level increased by 4 hours post-induction and decreased gradually up to 10 h post-induction in yeast expressing AtBI-1 alone, suggesting possible autoregulation. AtBI-1 mRNA accumulated at a higher level at 2h post-induction and stayed at a similar level up to 6 h post-induction in cells co-expressing PAP and either full-length or C-terminally deleted AtBI-1 (Fig. 6B). We conclude that AtBI-1 and PAP mRNA expression is upregulated in yeast cells expressing both proteins, consistent with the reduction in the cytotoxicity of PAP in these cells.

### AtBI-1 and PAP proteins are expressed

**Figure 7:**
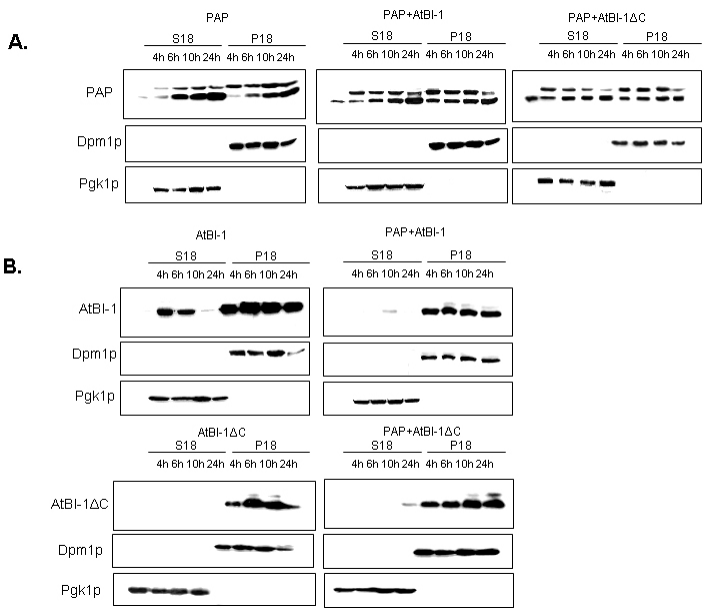
FIGURE 7: Immunoblot analysis. **(A)** PAP expression in yeast cells expressing AtBI-1 or AtBI-1ΔC. **(B)** AtBI-1 or AtBI-1ΔC expression in yeast cells expressing PAP. Yeast transformants were grown in SD-U-L glucose media. The expression of PAP and AtBI-1 was induced for indicated hours, lysed and fractionated into membrane (P18) and cytosolic (S18) components. The amount of 10 mg protein was separated on 15 % SDS-PAGE. Proteins were transferred into nitrocellulose membrane and probed with polyclonal PAP antiserum and monoclonal V5 antibody. The membrane marker Dpm1p and the cytosolic marker Pgk1p were used as controls to show equal amount of loading and lack of cross-contamination.

We then investigated PAP expression level in yeast extracts to determine whether the reduced cytotoxicity of PAP is due to altered expression or subcellular localization. We fractionated yeast extracts at various times after induction into cytoplasmic and membrane fractions 62 and analyzed each fraction for the presence of PAP and AtBI-1 proteins. ER membrane protein, Dpm1p and the cytosolic protein Pgk1p have been used as controls for fractionation. It has been already reported that both the precursor form of PAP and the mature form are associated with the ER membrane in yeast 63. As shown in Fig. 7A, PAP level was higher in both the membrane and the cytosol fraction at 4 and 6 h post-induction in yeast cells expressing PAP and AtBI-1 as compared to that of the cells expressing PAP alone. We analyzed AtBI-1 expression in both cytosolic and membrane fractions (Fig. 7B). AtBI-1 was detected in the cytosol and membrane fraction in cells expressing AtBI-1 alone. However, AtBI-1 was detected only in the membrane fraction in cells expressing AtBI-1 together with PAP (Fig. 7B). AtBI-1 and PAP mRNA expression did not correlate to protein levels in yeast cells expressing both proteins. This was previously observed by Di *et al. *64.

In contrast to full-length AtBI-1, we did not observe any AtBI-1∆C in the cytosolic fraction (Fig. 7B). When co-transformed with AtBI-1∆C and PAP, yeast cells slightly expressed AtBI-1∆C in cytosolic fraction by 24 hours post-induction (Fig. 7B).

### AtBI-1 binds to PAP *in vitro*

**Figure 8:**
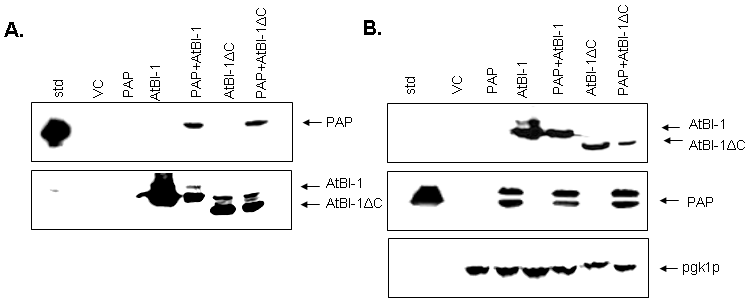
FIGURE 8: Co-immunoprecipitation assay. **(A)** Co-immunoprecipitation of PAP with either AtBI-1 or AtBI-1∆C. Total proteins isolated from yeast cells induced to express PAP, AtBI-1, AtBI-1∆C and vector control (VC) for 6 hours were incubated with V5-antibody and immunoprecipitated with protein A-Sepharose beads. Immunoprecipitated proteins were separated on 15 %SDS-PAGE, transferred to nitrocellulose and probed with affinity purified PAP antibody or with V5-antibody. **(B)** The total lysate from cells expressing PAP, AtBI-1 and AtBI-1∆C subjected to SDS PAGE/immunoblot analysis using V5 and PAP-antibody to show the level of expression of both proteins.

To examine the possibility that AtBI-1 may reduce the cytotoxicity of PAP by binding to it and forming a heterodimer, we used a co-immunoprecipitation assay in yeast cells expressing both PAP and AtBI-1. Total protein extracted from yeast co-expressing PAP and AtBI-1 were co-immunoprecipitated with the V5 monoclonal antibody. Total protein extracted from yeast expressing PAP alone and vector control was used as a negative control. PAP and AtBI-1 were co-immunoprecipitated with V5 antibody (Fig. 8A). Immunoblot analysis using total lysate was used to show the level of expression of both proteins (Fig. 8B). We next performed co-immunoprecipitation assay using AtBI-1∆C. These results indicated that AtBI-1 binds directly to PAP and C-terminal deletion of 23 aa did not change the binding capacity of AtBI-1 to PAP. These finding demonstrate that AtBI-1 can rescue yeast cells from cytotoxicity of PAP by binding it to form a heterodimer.

## DISCUSSION

We present evidence here that cells expressing PAP, a type I ribosome inactivating protein, exhibit nuclear fragmentation characterized by DAPI stained multiple regions in the nucleus with extensive vacuolization. These alterations were absent in cells expressing vector control, AtBI-1 and AtBI-1∆C (Fig. 1). The number of cells exhibiting nuclear fragmentation was decreased in cells expressing PAP and AtBI-1, indicating protective effect of AtBI-1 on PAP induced cell death. Besides inhibition of protein synthesis, PAP, a type I RIP, cleaves single-stranded 65 as well as double-stranded DNA 17 using the same active site required to depurinate rRNA. The cleavage of DNA in the nucleus and nuclear fragmentation are typical apoptotic features in yeast 66,67. It is tempting to think that the nuclear fragmentation in cell death induced by PAP is due to its nuclease activity. Similarly, some RIPs induce DNA damage by their nuclease activity 20,68. However, this aspect needs to be studied in depth to determine whether RIPs can enter the nucleus to induce DNA damage by nuclease activity and trigger apoptosis.

ROS production is involved in many types of cell death processes in animals, yeast and plants 55,59,60. Previous studies showed that ROS production is implicated in ricin-induced apoptotic cell death in mammalian cells as well as in yeast 28,69. Yeast cells exposed to oxidative stress or expressing mammalian Bax also induce ROS production 57. To determine if ROS is accumulated in yeast expressing PAP, intracellular ROS levels were quantified. The ROS accumulation in cells expressing PAP at 24 hours post-induction was almost 2-fold higher than that of vector control suggesting that ROS may act as an effector of apoptosis and trigger cell death signaling pathways. ROS induction precedes cell death in PAP expressing cells, suggesting that ROS may act as an effector of apoptosis and trigger cell death signaling pathways in those cells. In this study, we demonstrated for the first time that PAP, a type I RIP induces ROS production. Since ribosome depurination and translation inhibition are not always correlated with the cytotoxicity of PAP, ROS production in cells expressing PAP may be an important step, leading to PAP induced cell death.

We present the first evidence that the cytotoxicity of PAP is not only due to the depurination and translation inhibition but also to cell death in yeast.

AtBI-1 suppresses Bax induced cell death in plants, mammalian and yeast cells 41,44,70. Bax induced cell death in *Arabidopsis *protoplast system is inhibited by the overexpression of AtBI-1 through ROS independent processes 44. We investigated the possibility that AtBI-1 can inhibit PAP toxicity through ROS dependent processes. We did not observe any decrease in ROS production in cells expressing PAP and AtBI-1 or AtBI-1∆C, indicating that AtBI-1 functions as a negative regulator of PAP induced cell death independent of ROS accumulation or downstream of ROS, or both. In addition, the deletion of C-terminal region of AtBI-1 did not affect ROS accumulation in cells expressing both proteins suggesting that both proteins inhibit PAP toxicity via ROS independent pathway. These results correlated well with the cell viability assay. Yeast expressing PAP and AtBI-1 were able to grow on glucose containing medium after induction for 4 h in galactose containing media (Fig. 3). To determine the role of the C-terminal region of AtBI-1 in reduced cytotoxicity of PAP, we co-transformed yeast cells with PAP and AtBI-1∆C, and AtBI-1∆C was able to rescue yeast cells from cytotoxicity of PAP at the same level as AtBI-1. It was recently reported that the deletion of C-terminal region of AtBI-1 abolishes the ability of AtBI-1 to suppress Bax-induced cell death in yeast 43. The same authors demonstrated that the formation of coiled-coil structure in C terminus of AtBI-1 is essential for Bax-induced cell death inhibition in yeast. In our study, the deletion of 23 aa, which eliminated the predicted formation of coiled-coil structure, altered the level of AtBI-1 in the cytosol. The deletion of 23 aa at C-terminal region of AtBI-1 did not affect cell death suppression activity of AtBI-1 against PAP. Our results suggest that the C-terminal region of AtBI-1 may be critical for its transport to the cytosol. Since both AtBI-1 and AtBI-1∆C are associated with the ER, they may inhibit PAP associated with the ER membrane fraction in yeast. We conclude that the C-terminal region of AtBI-1 is not critical for the interaction between AtBI-1 and PAP, and the cell death inhibition activity of AtBI-1 against PAP. The structural model of Human Bax inhibitor-1 (hBI-1) revealed a 6-TM topology with both N- and C-termini in the cytoplasm and exhibits PH-sensitive calcium leak activities, proposed to be mediated by the C-terminal region 71. By homology, the C-terminal region of AtBI-1 may also have PH-sensitive calcium leak activity.

Hudak *et al.* 51 identified the PAP residues that are critical for ribosome depurination, inhibition of translation and cytotoxicity, and demonstrated that ribosome depurination is not sufficient for the inhibition of translation and cytotoxicity. Our results support this observation. Even though, rRNA depurination level was higher and translation inhibition was not affected in yeast expressing PAP and either AtBI-1 or AtBI-1∆C, cells were able to survive on galactose containing medium. These results show that AtBI-1 inhibited cell death caused by PAP in yeast independent of ribosome depurination and translation inhibition.

BI-1 was shown to be Bcl-2 binding but not Bax-binding protein with antiapoptotic activity 70. To further investigate a direct interaction between PAP and AtBI-1, we conducted co-immunoprecipitation assay with cells expressing both proteins. AtBI-1 as well as AtBI-1∆C proteins were able to bind to PAP at 6 hours post-induction. At 6 hours post-induction, both AtBI-1 and AtBI-1∆C were able to bind to the precursor form of PAP but not to mature PAP. The deletion of 23 aa at C-terminal region of AtBI-1 did neither abolish nor diminish the binding capacity of protein to PAP suggesting that the C-terminal region is not critical for this interaction.

Plant and animal BI-1 proteins are located mostly in the ER and the perinuclear region 38,41,70. Although the precursor form of PAP is mostly associated with the ER membrane, it is not exclusively localized in the ER 72. At 6 h post-induction we found the precursor form of PAP in cytosolic fraction as well as in the membrane fraction as described previously by Parikh *et al.* 72. However, AtBI-1 was only associated with the membrane fraction in yeast co-expressing PAP and AtBI-1, suggesting that the binding may take place in the ER.

Ricin inhibits adaptation responses to ER stress by preventing *HAC1* mRNA splicing and Ire1p signaling to downstream mediators of UPR 73. The inability to activate UPR in response to ER stress contributes to ricin-mediated cell death. By analogy with ricin, we can speculate that PAP may interfere with UPR therefore causing ER stress induced cell death. We recently showed that Bax expression induced the UPR in yeast and this was associated with *HAC1* mRNA splicing 74. Yeast cells deficient for yeast bax inhibitor (∆*bxi1*) are not only more sensitive to ER stress-inducing drugs but also have a decreased UPR 75. By homology with *BXI1, *AtBI-1 could also regulate PAP-induced cell death by UPR.

In summary, we show here that PAP induces cell death in yeast and AtBI-1 inhibits PAP induced cell death. We present evidence that the C-terminal region of AtBI-1 is not required to reduce PAP cytotoxicity. We demonstrate that AtBI-1 inhibits cell death induced by PAP independent of ribosome depurination and translation inhibition. Future experiments will characterize the mechanism by which AtBI-1 inhibits PAP cytotoxicity.

## MATERIALS AND METHODS

### Determination of cell viability by Evan`s blue staining, chromatin staining and ROS measurement

Cells were collected after induction at the times indicated, washed in PBS buffer and Evans blue was added to 1 ml of 0.6 OD cells at the concentration of 0.5% in PBS buffer and stained at room temperature for 30 min. After staining cells were washed several times with ddH_2_O to remove unbound dye from cultures before observation. Cells were counted using Zeis Axiovert 200 inverted microscope. The percentage of cell death was calculated by counting ~800 total cells as described by Xu *et al.* 71.

To detect nuclear fragmentation, yeast cells were washed with PBS buffer, fixed in 100% ethanol at room temperature for 5 min. and washed again. For nuclear staining, samples were incubated for 5 min. with 0.5 µg*ml^-1^ diaminopheylindole (DAP) in PBS and analyzed after washing by Zeiss Axiovert 200 inverted microscope with the epifluorescence setting (Axiovision 3.0; Carl Zeiss Vision GmbH).

Intracellular production of H_2_O_2_ was measured using the antioxidant sensitive probe 2’,7’-dichlorodihydrofluorescein diacetate (DCDHF-DA) (Invitrogen, Carlsbad, CA). 2 µl of fresh 5 mM DCDHF-DA was added to 1 ml of yeast culture (10^7^ cells) and incubated at 28°C for 45 min. The cells were then washed twice in sterile distilled water and resuspended in 1 ml of 50 mM Tris-HCl, pH 7.5. After 20 µl of chloroform and 10 µl of 0.1% SDS were added, the cells were incubated for 15 min. and pelleted. The fluorescence of the supernatant was measured using an HTS700 Perkin Elmer bioassay reader (Wellesley, MA) with excitation at 485 nm and emission at 525 nm.

### Plasmids

The cloning of PAP cDNA into NT198 under the control of *GAL1* promoter used in this study was described previously 7,73 . AtBI-1 (AB025927) was cloned into the yeast expression vector pYES2.1 (pYES2.1 TOPO TA expression kit, Invitrogen, USA) in upstream of V5 epitope by PCR using 5’GGATCCACGATGGATGCGTTCTCTTCCTTC3’ and 5’GTTTCTCC-TTTTCTTCTTCTTCTC3’ primers and into the pTKB175 without a tag. After the cloning, vectors were transformed into *E. coli* DH5α. The sequences were confirmed by sequencing two times using specific primers.

### Yeast transformation and cell viability

The *S. cerevisiae *strain W303 (*MAT***a ***ade2 trp-1 ura-3 leu2-3,112 his3-11,15 can1-100*) (from B. Thomas, Columbia university, New York, NY) was used for all transformations.

Cells were transformed and co-transformed as described previously 51. One-half of transformed yeast suspension was plated onto 2% glucose media, the other half was plated onto 2% galactose containing media. The toxicity of PAP was verified by re-plating the selected colonies onto both 2% glucose and 2% galactose media.

For cell viability, transformed and co-transformed yeast cells were grown on SD-Leu containing 2% glucose to an *A*_600_ of 0.3 and then transferred to selective medium containing 2% galactose to induce PAP and AtBI-1 expression. A serial dilution of cells was plated on selective media containing 2% glucose at 0, 4, 6, 10, and 12 h post-induction. Plates were incubated at 30°C for approximately 48 h.

### Growth conditions

Yeast cells were grown in YPD rich medium or synthetic dropout (SD) medium with appropriate amino acids at 30°C. Yeast cells transformed with PAP and AtBI-1 were grown initially at 30°C in a total volume of 100 ml of selective medium supplemented with 2% raffinose to a starting A_600_ of 0.6. Yeast cells were pelleted by centrifugation and washed with SD medium before replacing with 100 ml of selective medium containing 2% galactose to a starting A_600_ of 0.3. Then, 5 ml of culture were sampled for protein isolation, 10 ml of culture for RNA isolation and 1 ml for a growth reading (A_600_) at different times post-induction.

### Yeast protein expression analysis

Total protein extraction from frozen yeast cells collected at different times post-induction was extracted as described by Hudak *et al. *76. Samples were separated on 15% SDS-PAGE, transferred to nitrocellulose membrane (Roche) and probed with affinity purified anti-PAP polyclonal antibody (1:5000). The AtBI-1 and mutant forms of AtBI-1 proteins level were determined by using V5 monoclonal antibody (1:5000) that recognize V5 epitope at C-terminal of the protein. PAP, AtBI-1 and AtBI-1 mutants were visualized by chemiluminescence using the Renaissance kit (PerkinElmer Life Sciences). The blots were then stripped with 8 M guanidine hydrochloride for 30 min and reprobed with 3-phosphoglycerate kinase (Pgk1 p; Molecular probes) (1:10000) as an internal loading control.

For cell fractionations, protein from frozen yeast cells collected during various times post-induction was extracted as described by Frey *et al*. (2001). Briefly, after addition of low-salt (LS) buffer (20mM HEPES-KOH, pH 7.6, 100 mM potassium acetate, 5 mM magnesium acetate, 1 mM EDTA, 2mM DTT and 0.1 mM PMSF, yeast protease inhibitor cocktail (Sigma) and acid-washed glass beads (Sigma), cells were vortexed for 1 min and chilled for 1 min on ice for a total of 8 cycles. Crude lysates were spanned at 1200 g for 2 min. The same lysate was then centrifuged an additional 20 min at 18 000 g. The pellet was washed twice with ice cold water and resuspended in LS buffer. The supernatant and pellet fraction were stored at -80°C.

### RNA analysis

Total RNA was extracted from yeast using hot phenol 17. cDNA was synthesized from 1 μg of total RNA in a 20 μl reaction, containing 1 × first-strand buffer (Invitrogen), 40 U/μl RNA Guard RNase inhibitor (Promega, Madison, WI, USA), 0.5 μg poly d(T) oligonucleotide (Promega), 40 mM dNTPs and Superscript II (Invitrogen) reverse transcriptase. Quantification of transcript levels by real-time PCR analysis was performed using an ABI Prism 7000 Sequence Detection System using the manufacturers’ protocols. For quantitative PCR, the primers used were as follows:

PAP,* 5’-*gggtaagatttcaacagcaattca-3’* and *5’-caccactggcatccact-agct-3’*;*

G6PD 5’-CAGCAATGACTTTCAACATC-GAA-3’ and 5’-CCGGCAC-GCATCATGAT-3’;

AtBI-1, 5’-GTTGTGCTCTTGTGGCGTCTGC-3’ *and* 5’- TCAAGGG-GCCAACAGAAGCACCT-3’.

### *In vivo* [_35_S] Methionine Incorporation

Yeast cells were grown to an A_600_ of 0.6 in SD selective medium supplemented with 2% raffinose. Cells were then resuspended at an A_600_ of 0.3 in 2% galactose containing SD selective medium for 4-10 hours in order to induce either PAP, AtBI-1 or mutant forms of AtBI-1. At time zero, 35 methionine was added to cells growing on galactose. At the various times post-induction, 600 ml of yeast cells were taken for growth measurements and an aliquot of 800 ml were assayed for methionine incorporation in triplicate as described by Parikh *et al.* 2002. Briefly, the yeast were added to 200 ml of 100% trichloroacetic acid and incubated for 10 min on ice followed by 20 min at 70°C. The precipitate then filtered through 24-mm glass microfiber filters (VWR), washed with ice-cold 5% trichloroacetic acid followed by 95% ethanol. Filters were dried overnight and incorporation was quantified in a scintillation counter. The Cpm was normalized to the A_600_ reading.

### rRNA depurination Assay

Depurination of ribosomal RNA was performed by primer extension analysis in according to Hudak *et al.*^15^. 2 mg of total yeast RNA from transformants was incubated with (α-^32^P) ATP end labeled 5’ reverse primer (5’- AGCGGATGGTGCTTCGCGGCAATG-3’) complementary to 73 nt 3’ end of depurination site for depurination product and 5’ reverse primer ( 5’-TTCACTCGCCGTTACTAAGG-3’) specific to the 3’ end of yeast 25S rRNA as an internal control. The presence of depurination was observed by synthesis of a 73 nt extension product corresponding to the depurination site. An aliquot of 4 ml of extension product was separated on a 6% polyacrylamide/7 M urea denaturing gel and visualized and quantified on a PhosphorImager (Amersham Biosciences).

### Co-immunoprecipitation

PAP and AtBI-1 expressed *in vivo* yeast cells were co-immunoprecipitated with the monoclonal antibody against V5 epitope essentially as described by Otto and Lee 77. Total protein extracts from cells induced to express PAP, AtBI-1 at 6 h post-induction were used as substrate for immunoprecipitation with Protein A-Sepharose beads. Proteins were eluted from the beads with SDS sample buffer and visualized by immunoblot analysis using the antibodies to PAP and AtBI-1.

### Statistical analyses

The data were subjected to ANOVA test according to completely randomized factorial design. Differences between means were determined with Fisher’s Least Significant (LSD) test. P value of ≤ 0.001 was considered statistically significant. All values are presented as the mean of three independent experiments with the corresponding Standard Deviation (SD).
